# Outcomes of Different Surgical Interventions for Treating Trigeminal Neuralgia: A Review

**DOI:** 10.7759/cureus.66724

**Published:** 2024-08-12

**Authors:** Billy McBenedict, Wilhelmina N Hauwanga, Melvin Chun Yang Yau, Anna Pogodina, Gurinder Singh, Amro Abdelrahman, Anusha Thomas, Emmanuel S Amadi, Yee Siew Lim, Siymon Bispo, Bruno Lima Pessôa

**Affiliations:** 1 Neurosurgery, Fluminense Federal University, Niterói, BRA; 2 Family Medicine, Faculty of Medicine, Federal University of the State of Rio de Janeiro, Rio de Janeiro, BRA; 3 Neurological Surgery, Monash University Malaysia, Subang Jaya, MYS; 4 Medicine, University of Buckingham, Buckingham, GBR; 5 Medical Sciences, Specialized University of the Americas, Panama, PAN; 6 Medical Education, Hamad Medical Corporation, Doha, QAT; 7 Neurology, Christian Medical College and Hospital, Ludhiana, IND; 8 Internal Medicine, Hallel Hospital Port Harcourt, Port Harcourt, NGA; 9 Surgery, International Medical University, Seremban, MYS

**Keywords:** facial pain management, neurosurgery outcomes, pain relief, surgical interventions, trigeminal neuralgia

## Abstract

Trigeminal neuralgia (TN) is a debilitating condition characterized by severe facial pain. Various surgical interventions are employed to manage this condition, including microvascular decompression (MVD), percutaneous radiofrequency rhizotomy (PRR), glycerol rhizotomy, percutaneous balloon compression (PBC), and stereotactic radiosurgery such as Gamma Knife radiosurgery (GKRS). This review synthesizes the outcomes of these interventions to provide an understanding of their efficacy and associated risks. MVD, known for its high initial relief rates, shows substantial long-term effectiveness, with recurrence rates varying based on patient demographics and comorbidities. GKRS offers significant pain relief with a favorable adverse event profile; however, recurrence rates increase over time, necessitating repeat procedures for sustained efficacy. PBC demonstrates high initial success, but pain recurrence is common, especially in patients with atypical TN. PRR provides immediate relief with a manageable recurrence rate and is particularly suitable for elderly patients and those with comorbidities. Glycerol rhizotomy, a cost-effective procedure, yields comparable outcomes to other interventions but requires careful patient selection. This review highlights the importance of tailored treatment approaches based on individual patient profiles, emphasizing the need for precise diagnostic criteria and careful patient selection to optimize outcomes. Long-term follow-up and the potential for repeat interventions are critical considerations in managing TN surgically.

## Introduction and background

Trigeminal neuralgia (TN) manifests as transient, painful attacks within the trigeminal nerve distribution, affecting approximately 4-13 of 100,000 individuals annually. This condition induces moderate to severe facial pain, often triggered by various stimuli, and can severely incapacitate patients. The frequency of episodes varies widely, from multiple occurrences daily to only a few times annually, significantly compromising patients' quality of life [1]. TN arises from compression of the trigeminal nerve by various factors such as blood vessels, tumors, or other disorders. This compression triggers intense, shooting pain lasting from seconds to minutes. Patients often endure excruciating pain during routine activities like speaking, eating, or exposure to cold air. These painful episodes alternate with pain-free intervals, ranging from minutes to weeks. Typically, TN affects one side of the face (unilaterally), particularly near the trigeminal nerve's end in the jaw region. Consultation with a neurologist is advisable if the pain radiates to the upper forehead or cervical nerve distribution [2].

TN can significantly impact daily activities and contribute to depression. This rare condition, often initially mistaken for tooth pain, manifests as unilateral facial pain triggered by light touch. It is classified as TN with concomitant pain by the International Classification for Headache Disorders, necessitating the exclusion of other facial pain causes. While neurological examinations are typically normal, individuals may report sensory and autonomic symptoms, with subtle sensory loss observed in longer-standing cases [3]. 

Incidence and prevalence data, predominantly from the USA, indicate an annual incidence of 5.9 per 100,000 women and 3.4 per 100,000 men, with higher rates in women across all age groups, particularly in men over 80 years (45.2/100,000) [3]. The exact cause of TN remains unclear, though the ignition theory is commonly hypothesized. Peripheral and central mechanisms, along with potential alterations in trigeminal nerve microstructure, may be involved. TN is more prevalent in individuals with multiple sclerosis (MS) and stroke. Hypertension poses a risk factor, particularly in women (relative risk (RR) 2.1, 95% CI 1.2-3.4), although evidence is less conclusive for men [3]. TN primarily stems from blood vessel compression on the trigeminal nerve root at the brain stem, yet conditions like MS, especially in young patients, are also linked to its development [1]. This condition affects various segments of the trigeminal nerve, which innervates the forehead, cheeks, and lower jaw via its maxillary, ophthalmic, and mandibular divisions [1]. While TN predominantly affects females after age 50, it can manifest unilaterally, with the right side more commonly affected, though bilateral involvement has been reported [1].

Characterized by electric shock-like sensations, TN's unpredictable and intense pain significantly impacts both physical and mental health, profoundly affecting patients' overall quality of life [4]. Even routine activities such as washing, shaving, smiling, or speaking can trigger these painful episodes [5]. TN presents as recurrent, unilateral, short-lasting pain attacks affecting one or more branches of the trigeminal nerve. The pain is described as sharp, lancinating, or electric-like, often accompanied by tic-like facial muscle spasms, earning it the nickname "tic douloureux." Attacks can be triggered by innocuous stimuli like talking or touching the face, occurring multiple times daily and worsening in frequency, duration, and intensity over time.

The etiology of TN remains elusive, although most cases stem from compression of the trigeminal nerve root near its entry into the pons. This compression, often accompanied by demyelination of sensory fibers within the nerve root or entry zone, is primarily attributed to vascular compression by an anomalous artery or vein, accounting for 80-90% of idiopathic TN cases. Less commonly, other compressive factors, such as benign posterior fossa tumors like acoustic neuroma, meningioma, and epidermoid cysts, can contribute to the condition [6]. TN has been associated with 25% of suicide attempts, with medication side effects exacerbating psychological distress and impacting social and occupational functioning. While randomized controlled trials on cognitive-behavioral therapies for TN are scarce, evidence from general pain studies suggests their potential efficacy [7].

For patients with persistent pain or those unable to tolerate medication due to side effects, several surgical interventions are possible. These encompass microvascular decompression (MVD), percutaneous radiofrequency rhizotomy (PRR), percutaneous glycerol rhizotomy, percutaneous balloon compression (PBC), and stereotactic radiosurgery (SRS), such as Gamma Knife radiosurgery (GKRS) or Cyberknife. However, the effectiveness and risks associated with these procedures vary [8].

MVD involves a suboccipital craniotomy to alleviate trigeminal nerve compression, offering highly effective pain control with initial relief rates between 80.3% and 96%, as established by Jannetta et al. [9,10]. GKRS, another treatment for refractory facial pain such as TN, achieves complete pain relief in approximately 70% of patients, with maintenance rates of 40-55% at three years and 25% at 10 years [11]. It is known for a favorable adverse event profile, primarily causing sensory changes such as facial numbness. PBC of the trigeminal ganglion, introduced in 1983, disrupts nerve signal transmission through controlled injury, a technique evolving from procedures developed in the 1950s [12]. PRR involves accessing the Gasserian ganglion with a needle to induce injury via heat, chemicals, or mechanical compression, with initial response rates of 97.6-99% [13,14], six-month response rates of 83.3-89.9% [13,15], and varied recurrence rates from 38.2% at one year to 10% at 6.5 years, with 41% maintaining complete pain control after 20 years [13].

## Review

Material and methods

The review used the methods outlined in the Preferred Reporting Items for Systematic Reviews and Meta-Analyses (PRISMA) guidelines for the organization and reporting of its results. A bias assessment was not performed because this study is not a systematic review.

Source Information and Search Strategy

An electronic search was performed across multiple research databases, including PubMed, Embase, Scopus, Web of Science, and Cochrane (Table 1). All databases were accessed on May 16, 2024. The search spanned the period between January 2014 and May 2024.

**Table 1:** Summary of the search strategy from the databases.

Database	Search strategy	Filters used
PubMed	(("Trigeminal Neuralgia"[Title/Abstract] OR "Trifacial Neuralgia"[Title/Abstract] OR "Tic Douloureux"[Title/Abstract])) AND (("Surgical Interventions"[Title/Abstract] OR "Surgery"[Title/Abstract] OR "Operative Procedures"[Title/Abstract]) AND ("Outcomes"[Title/Abstract] OR "Results"[Title/Abstract] OR "Efficacy"[Title/Abstract]))	Humans only, English language, exclude preprints, filter years 2014-2024
Embase	'trigeminal neuralgia':ab,ti AND ('surgical interventions':ab,ti OR 'surgery':ab,ti OR 'operative procedures':ab,ti) AND ('outcomes':ab,ti OR 'results':ab,ti OR 'efficacy':ab,ti)	Humans only, English language, filter years 2014-2024
Scopus	TITLE-ABS-KEY ( "Trigeminal Neuralgia" OR "Trifacial Neuralgia" OR "Tic Douloureux" ) AND ( "Surgical Interventions" OR "Surgery" OR "Operative Procedures" ) AND ( "Outcomes" OR "Results" OR "Efficacy" )	Humans only, English language, filter years 2014-2024
Web of Science	(AB=("Trigeminal Neuralgia" OR "Trifacial Neuralgia" OR "Tic Douloureux")) AND AB=("Surgical Interventions" OR "Surgery" OR "Operative Procedures")) AND AB=("Outcomes" OR "Results" OR "Efficacy")	Humans only, English language, filter years 2014-2024
Cochrane	("Surgical Interventions" OR "Surgery" OR "Operative Procedures"):ti,ab,kw AND ("Outcomes" OR "Results" OR "Efficacy"):ti,ab,kw AND ("Trigeminal Neuralgia" OR "Epileptiform Neuralgia" OR "Trifacial Neuralgia" OR "Tic Douloureux" OR "Fothergill Disease"):ti,ab,kw	Humans only, English language, filter years 2014-2024

Inclusion and Exclusion Criteria

The inclusion criteria covered studies involving outcomes of different surgical interventions for treating TN in adult humans. Eligible study designs included primary research studies published in English. Clinical cases were only included if they involved more than 10 participants. Studies that report the follow-up of patients (followed or monitored for a period of time) who underwent surgical interventions (MVD, GKRS, balloon compression, glycerol rhizotomy, or radiofrequency thermal injury). Only peer-reviewed journal articles in English were considered for inclusion. Exclusion criteria covered animal, pediatric, non-TN articles, non-peer-reviewed articles, systematic reviews, reviews, conference abstracts, and editorials.

Results

**Figure 1:**
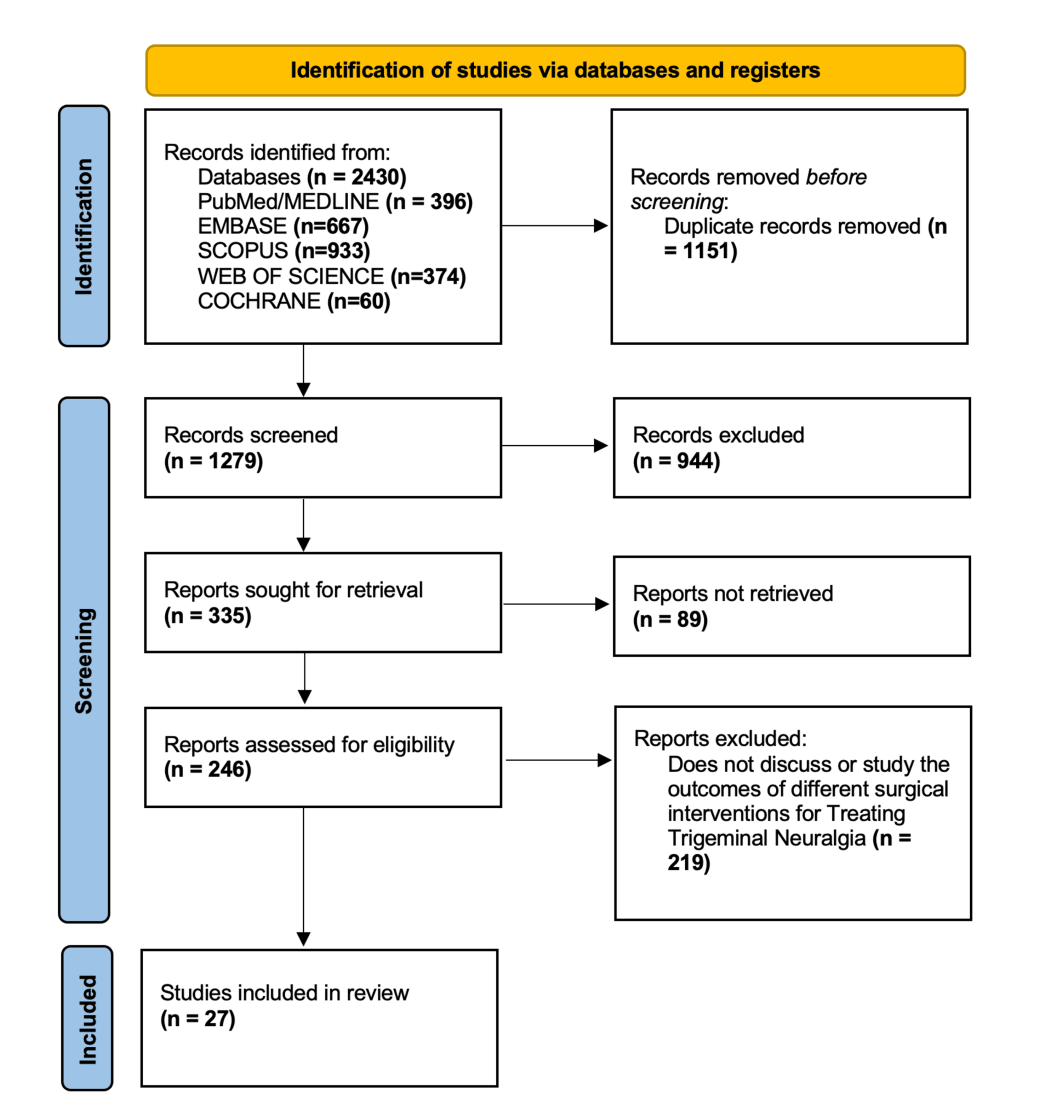
PRISMA flow diagram showing the steps taken to filter the articles for this review. PRISMA: Preferred Reporting Items for Systematic Reviews and Meta-Analyses.

Through our search strategy, we identified a total of 2430 articles (Figure 1), comprising 396 from PubMed/Medline, 667 from Embase, 933 from Scopus, 374 from Web of Science, and 60 from Cochrane. Filters were applied based on the inclusion/exclusion criteria. The articles were transferred to an Excel sheet, where 1151 duplicates were manually removed, resulting in 1279 articles. These 1279 articles were further scrutinized based on their titles and abstracts, leading to the disqualification of 944, leaving 335 articles. Full texts for 89 articles could not be retrieved, leaving us with 246 papers for eligibility assessment. After a thorough full-text review, 219 papers were excluded, resulting in 27 articles being included/used to synthesize this review.

Discussion

GKRS: Primary Outcomes and Recurrence Rates

GKRS has shown promising results in providing pain relief for patients, with various studies documenting significant rates of initial and sustained pain relief over different follow-up periods. A study observed that the average onset of pain relief following GKRS occurred at 2.2 months, with a cure rate of 53.6% and an improvement in 35.7% of patients [16]. It was reported that a recurrence rate led to 10.7% of cases being classified as ineffective treatments [16]. It was reported that GKRS is highly effective for elderly patients, with a cure rate of 53.6% and an improvement rate of 35.7% [16]. Facial numbness and dysesthesia were observed in 12.5% of patients following GKRS [16]. Facial numbness was recorded as a common side effect of GKRS, often associated with improved long-term pain control. Additionally, it was documented that 46.8% of patients achieved complete pain relief at Barrow Neurological Institute (BNI) within an average of 30 days [17]. In a study of 503 patients, 89% experienced initial pain relief after GKRS, with 73% maintaining significant pain relief (BNI scores I-IIIa) at one year, 65% at two years, and 41% at five years. Additionally, it was noted that 78.7% of patients did not require additional surgical interventions post-GKRS, suggesting a low recurrence rate [17]. It was found that 82.5% of patients experienced good outcomes, with 46.8% achieving complete pain relief (BNI I) [17]. The study indicated a 4.2% incidence of facial numbness, with no significant cases of radiation necrosis, edema, or diplopia [17].

In a study, identified post-GKRS complications included facial sensory dysfunction in 10.5% of patients, with reports of new or increased facial numbness. Notably, patients experiencing sensory loss often had better long-term pain control [18]. Including cases with adequate pain control (BNI scores I-IIIb), relief rates were 80% at one year, 71% at three years, 46% at five years, and 30% at 10 years [18]. It was reported that 89% of patients achieved initial pain relief post-GKRS, with significant pain relief (BNI scores I-IIIa) sustained in 73% at one year, 65% at two years, and 41% at five years. Including those with adequate pain control (BNI scores I-IIIb), the rates were 80% at one year, 71% at three years, 46% at five years, and 30% at 10 years [18]. Pain recurrence occurred in 193 patients, with a median time to recurrence of 48 months. Factors contributing to earlier recurrence included additional symptoms and a history of multiple failed surgeries [18]. It is recommended that patients should be informed about the high likelihood of significant pain relief, especially in the initial years following GKRS. It is important to also highlight the possibility of some pain recurrence and the availability of repeat procedures if necessary. Educating patients on the long-term pain relief rates and the options for additional GKRS or other interventions in case of pain recurrence is essential [18].

Similarly, it was found that favorable pain relief was observed in patients with a median follow-up of 11 months. Diffusivity metrics at three months post-GKRS correlated with long-term pain relief, suggesting an early predictor of success [19]. A study of a smaller cohort of 16 patients observed favorable pain relief at a median follow-up of 11 months. Significant improvements were noted in patients who responded well to GKRS at three months post-procedure [19]. Additionally, no severe complications were reported, although 31% of participants experienced facial numbness, which did not significantly impact the overall positive outcomes in pain relief [19]. It was also reported that 75% of patients were pain-free post-GKRS, with a median time to pain recurrence of 53 months, highlighting the procedure's effectiveness and the need for long-term follow-up [20]. GKRS has shown promising results in the treatment of patients with chronic pain, as evidenced by multiple studies. A prospective study found that 75% of patients were pain-free post-GKRS, with a median time to pain recurrence of 53 months, demonstrating the consistency of GKRS with other large series and underscoring the importance of long-term follow-up [20]. Furthermore, it was observed that the median time to pain recurrence was 53 months post-GKRS, highlighting the necessity for extended monitoring to evaluate the long-term effectiveness of pain relief [20]. Sensory deficits were significantly associated with favorable pain outcomes post-GKRS, suggesting a trade-off between efficacy and side effects [20]. In comparison, a study on microsurgery versus SRS for small petrous apex meningiomas indicated that while GKRS is effective, microsurgery may be preferred for specific anatomical considerations [21]. The high success rates, consistent with other studies, in relieving TN symptoms were reaffirmed [22].

GKRS has been associated with high levels of patient satisfaction due to its significant pain relief and minimal invasive complications, leading to notable improvements in quality of life, highlighting improvements in quality of life, especially among elderly patients who are often at higher risk with more invasive surgical options [16,17,20]. It was found that most patients expressed high satisfaction with their quality of life post-GKRS, despite some experiencing sensory disturbances. Notably, a majority of patients with persistent sensory dysfunction reported improved quality of life after treatment, indicating a favorable balance between pain relief and manageable side effects [18]. Using the BNI scale, 73% of patients experienced significant pain relief (BNI Scores I-IIIa) at one year, with 46% maintaining this relief at five years [18]. Similarly, patients with favorable responses reported significant pain relief (BNI Scores I-III) at the last follow-up [19]. Another study using the BNI Pain Scale reported a 46.8% complete pain relief (BNI score of I) within an average of 30 days [17]. The BNI Pain Intensity Scale, which categorizes pain from I (no pain) to V (severe pain/no pain relief), is extensively used to quantify the level of pain relief following GKRS and plays a crucial role in comparing patient outcomes across various studies. Pain scales were applied to evaluate outcomes in another study, although specific details of the measures were not included in the summary [22]. Additionally, the potential of imaging biomarkers to predict treatment responses and tailor therapeutic strategies early in the treatment course was underscored by investigating changes in diffusion tensor imaging (DTI) metrics as a diagnostic tool, linking these changes to clinical outcomes [19].

Patients with no prior surgical history and a shorter duration of TN symptoms before GKRS show better outcomes. It was found that patients who had not undergone previous surgical interventions and those who had experienced pain for less than three years achieved quicker and more durable pain relief [18]. Additionally, the presence of preoperative sensory disturbances was associated with improved pain control post-GKRS. Such pre-existing sensory disturbances indicate a more favorable prognosis for pain relief [19]. Elderly patients, especially those over 80, are ideal candidates for GKRS due to its non-invasive nature and lower complication rates compared to more invasive procedures like MVD [16]. A patient's history of prior surgical interventions does not significantly affect the efficacy of GKRS, underscoring its broad applicability even for patients with previous surgical treatments [17]. The importance of precise diagnostic criteria and careful patient selection to optimize treatment outcomes are relevant.

The proficiency of the medical team performing GKRS significantly impacts patient outcomes. Research has demonstrated that experienced practitioners achieve better pain control and fewer complications due to their expertise in precise targeting and dose planning [18]. It has been highlighted that precision in targeting the trigeminal nerve root entry zone is crucial for GKRS, with typical doses ranging from 35 to 40 Gy. While specific details on practitioner experience are not explicitly outlined, the studies suggest that a strong background in neurosurgery and radiology is essential for successful outcomes. GKRS involves meticulous planning and execution, often requiring the collaboration of multidisciplinary teams to ensure optimal patient care [16,17].

Younger patients generally experience better outcomes with GKRS due to fewer comorbidities and better overall health. Research has shown that younger patients with a shorter duration of symptoms before surgery tend to have more favorable outcomes [18]. Conversely, elderly patients, especially those over 80, also tend to have better outcomes due to the high safety profile of GKRS. Studies indicate that older age does not negatively impact the effectiveness of GKRS, making it a suitable option for older adults [16,17]. Patients with classic TN respond better to GKRS compared to those with atypical or secondary forms of TN. Significant pain relief was observed in classic TN patients post-GKRS [19]. Additionally, preoperative sensory disturbances such as numbness are associated with better pain control post-GKRS, with patients having pre-existing sensory disturbances more likely to experience significant pain relief [19]. Patients with clear vascular compression as the underlying cause of TN typically experience better outcomes, while the presence of MS or other complicating factors can influence the effectiveness of GKRS, necessitating tailored treatment approaches [16].

MVD: Primary Outcomes and Recurrence Rates

MVD has been shown to achieve significant pain relief for the majority of TN patients. In a study, 93.18% of patients experienced complete pain relief at discharge, with 15.66% developing recurrent pain during a mean follow-up of 8.9 years [23]. Additional treatments, such as pharmacologic therapy, SRS, and percutaneous rhizotomy (PR), provided adequate pain control for many failed cases, though 6.12% had persistent pain despite various treatments. Persistent facial sensory dysfunction was observed in 4.47% of patients, with hearing impairment in 0.94%, minor wound infections in 2.12%, and cerebrospinal fluid (CSF) leakage in 0.47%, sometimes requiring surgical repair. One patient developed a bilateral frontal epidural hematoma, which was successfully removed, while another suffered a fatal cerebellopontine angle hematoma [23].

A study highlighted racial disparities, with Black or African American patients reporting higher postoperative recurrence rates (41.7%) compared to White patients (25.1%) and experiencing shorter pain-free durations [24]. A larger proportion of Black or African American patients were at nearly 50% greater risk for pain recurrence. It was reported that patients who underwent MVD as a second-line treatment had a longer pain-free duration compared to those undergoing repeat SRS. Immediate pain relief was reported in 95.7% of patients, with 94.2% pain-free at the first follow-up after three months. Temporary sensory changes post-SRS were noted, but no permanent numbness or dysesthesias were reported before second procedures [25].

In a study, long-term follow-up indicated that patients aged 70 or older had a tendency towards higher recurrence rates [26]. Long-term deficits included moderate hearing impairment in a few patients and persistent hypoesthesia in six patients. Chronic headaches and severe vertigo were rare, with no significant differences in persistent neurological deficits between age groups and no treatment-related mortality [26]. MVD was the primary surgical treatment for 96.1% of patients under 70 and 86.2% of those 70 and older, with similar surgery and hospital stay durations. Transient neurological deficits were low, with mild facial hypoesthesia being the most common [26]. Another study reported that initial pain relief was achieved in 80% of patients at discharge, but pain recurred in 75% within a median of four months [27]. It was observed that 83.3% of patients experienced complete pain relief immediately after MVD, and 72.2% of them maintained this relief at the last follow-up [28]. Post-MVD, no fatalities or major morbidities were reported, with 19.4% experiencing new or worsened facial numbness, 8.3% reporting bothersome sensory loss, and 2.8% with CSF leakage resolved via lumbar drainage [28]. The risk of facial numbness was higher in patients with prior GKRS [28].

Long-term pain-free rates were documented at 94.1% at one year and 83.0% at 10 years, with postoperative complications including minor wound infections and aseptic meningitis [29]. Additionally, it was found that patients requiring additional procedures typically achieved pain relief after multiple interventions, with the average number of procedures per patient being higher in the radiofrequency (RF) cohort [30]. Facial numbness was reported in 16% of RF MVD patients, 50% of patients, and 36% of SRS patients, although numbness was not necessary for pain relief. The MVD cohort experienced a 9% complication rate, including CSF leakage (two patients), presumed meningitis (two patients), pulmonary embolism (one patient), and wound infections (two patients), all of whom fully recovered [30]. 

It was reported that 83.4% of patients achieved long-term pain relief (BNI I-III) after MVD, although 9% developed refractory recurrent pain [31]. Complications included CSF leakage in 7% and wound infections in 5%. One patient developed permanent hypoacusia, and another required urgent surgery for a cerebellar hematoma, fully recovering. The complication rates between older and younger patients were similar, with no significant difference in neurological or medical complications [31]. A cohort of 40 individuals (59% female, average age 54.26 years) underwent MVD [32]. Preoperatively, 61.76% had a BNI pain score of 4, and 55.6% had a score of 5. Postoperatively, 50% achieved a BNI score of 1 or 2, indicating full recovery without medication, while 14.71% had a score of 3 or 5, indicating no recovery. Regarding complications, 9% experienced CSF leaks, 6% had wound infections, and 3% suffered hearing loss [32]. A total of 80% of participants were satisfied with their outcomes, and 84% of TN patients were satisfied post-MVD [32].

MVD shows superior long-term outcomes compared to other surgical treatments, making it a preferred option, especially for older patients [26]. Kaplan-Meier survival curves show that MVD has cure rates of 89.3% at one year, 80.5% at three years, and 71.2% at eight years, outperforming PR and SRS [23]. However, in cases with poor response to MVD, percutaneous procedures might be more appropriate [27]. Racial and socioeconomic factors influence pain presentation and outcomes, emphasizing the importance of patient-physician trust and patient involvement in treatment decisions. Environmental risk factors and race may affect pain outcomes, suggesting a need for further investigation into their impact on postsurgical results [24]. Decision-making between MVD and repeat SRS should consider patient preferences, medical comorbidities, and magnetic resonance imaging (MRI) evidence of vascular compression. In a study, univariate ordinal regression analysis indicated that total surgical duration, previous rhizotomy, prior MVD, patient income, and the time between symptom onset and neurosurgical visit or between neurosurgical visit and MVD did not predict worse pain outcomes at final follow-up [24].

Glycerol Rhizotomy: Outcomes

Percutaneous retrogasserian glycerol rhizotomy (PRGR) is a simple, safe, and cost-effective procedure that does not require expensive equipment, offering outcomes comparable to other, more costly open and minimally invasive procedures [33]. Percutaneous radiofrequency thermocoagulation is strongly recommended for patients with intractable TN and those who cannot tolerate medications due to its simplicity, safety, and cost-effectiveness, with minimal complications. It demonstrates high efficacy, with recurrence rates comparable to those of other procedures [33]. A study that included 93 patients with typical TN who successfully received glycerol injections recorded immediate pain relief in 96.8% of patients, and long-term pain relief was maintained in 89.4% of patients. Three patients (3.2%) experienced continued pain with reduced intensity. Pain recurrence occurred in 10.4% of patients (eight cases) [33].

Studies have investigated the effectiveness and outcomes of different rhizotomy techniques for pain management. In a cohort study, 21 patients with 26 surgically treated sides underwent their first rhizotomy, including 13 GKRS procedures and 13 percutaneous rhizotomies (10 glycerol injections and three balloon compressions). Initial pain relief, defined as BNI scores of 1-3, was achieved in 12 of 13 (92%) sides treated with GKRS and 10 of 13 (77%) sides treated with PR [34]. In a study using PRGR, excluding technical failures, the initial success rate was 85%, with a median pain-free duration of 21 months. At the last follow-up, 47 patients (38%) remained pain-free. Eight procedures (6%) in the PRGR group were preceded by technically failed attempts. The median duration of pain relief was 21 months after PRGR and 20 months after PBC. Both methods carried a high risk of hypesthesia/hypalgesia (p<0.001), which partially reversed over time [35]. 

A study investigated glycerol rhizotomy as a potential future treatment for TN resistant to medical treatment. The study included 74 patients who underwent MVD and received a direct glycerin injection into the inferior third of the cisternal portion of the trigeminal nerve [36]. These patients were compared to a control group of 526 patients who only underwent MVD. The average follow-up period for the 74 patients who received both MVD and glycerin injection was 19.1 ± 18.0 months. Of these patients, 33 (44.6%) had failed a prior TN intervention. The BNI Pain Intensity scores improved on average from 4.1 ± 0.4 before surgery to 2.1±1.2 after surgery. A total of 95.9% of patients documented improvement in pain following the surgery. Some patients experienced scorching pain post-surgery, and other minor complications such as hearing loss, facial palsy, CSF leak, and incisional infection were recorded [36].

Balloon Compression: Primary Outcomes and Recurrence Rates

PBC for MS-associated TN provides significant initial and long-term pain relief, despite some requiring repeat procedures. The results of PBC treatment in 222 patients after both the initial and final procedures showed that 89% of patients were pain-free following their first procedure. Despite the recurrence of pain in 69 patients necessitating repeat procedures, 88% of all patients remained pain-free at their last follow-up. Among the 69 patients who underwent repeated PBC, 93% were pain-free after the initial procedure, and 90% experienced excellent or good pain relief after the final procedure. The proportions of patients with excellent results after their first PBC procedure were analyzed in relation to demographic and clinical variables, revealing no significant relationship with age, gender, symptom duration, or previous surgery. However, patients with atypical pain were less likely to achieve excellent results compared to those with typical pain (61.4% vs. 82.9%, p<0.001) [37].

Recurrence rates after PBC for TN show that at least 46% of patients experience recurrent pain, and prior surgery significantly increases the likelihood of recurrence. A study found that a total of 103 patients (46%) experienced recurrent trigeminal pain after PBC. The estimated recurrence-free rates of TN after PBC were 47.6% at one year, 34% at two years, 18.5% at three years, and 8.7% at four years. Pain most frequently recurred 12 months after surgery, with a median of 12 months and a range from one day to 180 months. Patients with atypical pain and longer symptom duration did not appear to be at a higher risk of recurrence compared to other patients (p=0.10, p=0.81). No statistically significant associations were found between recurrence and age (p=0.06), sex (p=0.17), or pain location (p=0.80). However, there was a higher rate of recurrence in patients with prior surgery (p=0.02) [37].

Another study reported that out of 16 patients, eight (50%) experienced a recurrence of their pain, with a mean interval to recurrence of 13.50±15.26 months. Among these eight patients, two (patients 6 and 7) had their pain controlled by medication, eliminating the need for additional surgery. The remaining six patients (patients 1, 3, 5, 11, 13, and 14) required one additional PBC. At the latest follow-up examination, an excellent outcome (BNI grade 1 or 2) was achieved for 12 of the 16 patients (75%), while a good outcome (BNI grade 3) was observed in four patients (25%). No patient was experiencing uncontrolled pain [38].

In a study, subgroup analyses of repeated treatments revealed that the time to recurrence after a fourth or fifth PBC treatment was significantly shorter compared to the first treatment, with median times to recurrence being 0 months versus 18 months, respectively. Additionally, patients who underwent three or more treatments had significantly worse outcomes from their first treatment compared to those who were treated only once or twice [39]. 

In a retrospective cohort study, 222 patients (142 females) were included, with a median age of 68 years (range: 25-97 years). Preoperative MRI imaging with gadolinium enhancement, and in some cases MRI angiography, was conducted to exclude intracranial pathology. None of the patients were found to have an associated brain tumor or vascular malformation [37]. The median duration of pain before undergoing PBC was 60 months. Participants were followed up for a median period of 31.1 months (range: less than one month to 202.7 months). Pain involving multiple divisions of the trigeminal nerve was reported in 160 patients (72.1%), with 121 patients (54.5%) undergoing procedures on the right side. Most patients (68.5%) experienced typical TN1, while 31.5% had other types of trigeminal pain. A significant portion (31.1%) underwent more than one procedure, totaling 320 PBCs performed at an average of 1.4 procedures per patient. In a case series, 10 patients with detectable lesions in the trigeminal pathway were studied. Factors such as affected trigeminal divisions, presence of preoperative deficits, and history of previous surgery did not significantly affect clinical outcomes. However, surgical observations like a pear-like balloon shape during the procedure and MRI-detectable lesions in the trigeminal pathway were associated with longer progression-free survival [38].

Radiofrequency Thermal Lesioning: Primary Outcomes and Recurrence Rates

In a study, 12 patients reported satisfactory pain relief post-percutaneous RF-trigeminal rhizotomy (TR), with seven experiencing immediate pain cessation and discontinuing medical therapy. Three patients occasionally required low-dose medications for pain management post-treatment [40]. During the follow-up period, one patient experienced recurrence 14 months post-treatment and achieved pain relief with repeated RF-TR guided by neuronavigation with MRI/intraoperative computed tomography (CT) fusion images. Intraoperative images revealed a failure of puncture into the trigeminal cistern due to a bony structure obstacle, with the needle positioned only 3.05 mm above the foramen ovale [40]. Real-time neuronavigation integrated with MRI/CT, alongside functional imaging of the trigeminal ganglion (based on anatomical topography derived from MRI/DTI), holds promise as a potential advancement in percutaneous procedures. This approach could enable more precise targeting and potentially lead to improved long-term outcomes [40].

A study found that after the second percutaneous radiofrequency rhizotomy (PRR) procedure, 30 patients (90.9%) experienced immediate pain relief (to an intensity ≤ BNI grade III), while three patients (9.1%) had persistent symptoms [41]. Excellent outcomes were defined as achieving BNI grade I, indicating no pain and no medication use. Good outcomes indicated occasional pain not requiring medication (BNI grade II) or moderate pain adequately controlled with medication (BNI grade III). Among those with immediate pain relief, 19 patients (63.4%) maintained excellent pain control throughout the follow-up period (mean 44.2 months, range 13-67), gradually discontinuing medications over several weeks. However, 11 patients (36.6%) who achieved pain relief after the second PRR experienced symptom recurrence (mean time to recurrence 17.1 months, range 5-82) [41]. All patients exhibited mild-to-moderate facial numbness following repeated PRR, which gradually alleviated. However, persistent numbness was reported in 30 patients (90.9%) during follow-up. Notably, two patients who reported no numbness during follow-up experienced pain recurrence. Post-procedural complications included masseter weakness observed in three patients (9.1%) and limited mouth opening in one patient (0.3%) [41].

In a study reporting surgical procedure comparative data over 19 years, 393 procedures were performed on 210 TN patients, with follow-up extending over 17 years through patient records and telephone follow-up [42]. The initial rates of complete pain relief, with or without medication, were 72% for glycerol, 80% for thermocoagulation, and 86% for PBC. PBC provided significantly longer relief than the other two procedures. The complication rates for glycerol, thermocoagulation, and PBC were 30.3%, 27.1%, and 43.5%, respectively. Although PBC is more likely to result in numbness and complications, these complications are largely minor and transitory [42].

*Limitations of the study* 

This study has some limitations. The retrospective nature of the included studies introduces potential biases related to patient selection and recall, limiting the ability to establish causation and affecting the robustness of the findings. Some studies had notably short follow-up periods, making it challenging to assess long-term efficacy and recurrence rates. The absence of randomized controlled trials (RCTs) in these reviews diminishes the strength of the evidence. Future research should aim to include RCTs to provide higher-quality evidence and enable more reliable comparisons of the treatment modalities.

## Conclusions

This review discussed the varying effectiveness and safety profiles of surgical interventions for treating TN. MVD consistently demonstrated high initial pain relief rates, with long-term efficacy maintained in a significant proportion of patients. The complication rates were relatively low, making MVD a preferred option, particularly for patients with clear vascular compression. GKRS also provided substantial pain relief with the added benefit of being minimally invasive, though some patients experienced sensory disturbances. Percutaneous procedures, including glycerol rhizotomy, balloon compression, and radiofrequency thermal lesioning, offered immediate pain relief with varying long-term outcomes. Balloon compression showed high initial success, though repeat procedures were often necessary due to recurrence. Glycerol rhizotomy and radiofrequency thermal lesioning were noted for their simplicity and cost-effectiveness, but the risk of sensory complications was notable. These findings underscore the importance of individualized treatment plans, considering patient-specific factors such as age, comorbidities, and anatomical considerations, to optimize clinical outcomes and enhance the quality of life for TN patients.
